# An *rbc*L mRNA-binding protein is associated with C_3_ to C_4_ evolution and light-induced production of Rubisco in *Flaveria*

**DOI:** 10.1093/jxb/erx264

**Published:** 2017-08-08

**Authors:** Pradeep Yerramsetty, Erin M Agar, Won C Yim, John C Cushman, James O Berry

**Affiliations:** 1 Department of Biological Sciences, State University of New York, Buffalo, NY, USA; 2 Department of Biochemistry and Molecular Biology, University of Nevada, Reno, NV, USA

**Keywords:** Cell type specificity, light regulation, nuclear-encoded mRNA-binding protein, phylogeny, plastid-encoded *rbc*L gene, post-transcriptional control, protein synthesis, RLSB and C_4_ evolution, Rubisco regulation, tissue specificity

## Abstract

Nuclear-encoded RLSB protein binds chloroplastic *rbc*L mRNA encoding the Rubisco large subunit. RLSB is highly conserved across all groups of land plants and is associated with positive post-transcriptional regulation of *rbc*L expression. In C_3_ leaves, RLSB and Rubisco occur in all chlorenchyma cell chloroplasts, while in C_4_ leaves these accumulate only within bundle sheath (BS) chloroplasts. RLSB’s role in *rbc*L expression makes modification of its localization a likely prerequisite for the evolutionary restriction of Rubisco to BS cells. Taking advantage of evolutionarily conserved RLSB orthologs in several C_3_, C_3_–C_4_, C_4_-like, and C_4_ photosynthetic types within the genus *Flaveria*, we show that low level RLSB sequence divergence and modification to BS specificity coincided with ontogeny of Rubisco specificity and Kranz anatomy during C_3_ to C_4_ evolution. In both C_3_ and C_4_ species, Rubisco production reflected RLSB production in all cell types, tissues, and conditions examined. Co-localization occurred only in photosynthetic tissues, and both proteins were co-ordinately induced by light at post-transcriptional levels. RLSB is currently the only mRNA-binding protein to be associated with *rbc*L gene regulation in any plant, with variations in sequence and acquisition of cell type specificity reflecting the progression of C_4_ evolution within the genus *Flaveria*.

## Teaser

RLSB occurs in photosynthetic tissues and is post-transcriptionally regulated by light. Modification to bundle sheath specificity correlated with minor sequence divergence and the ontogeny of Kranz development during C_4_ evolution

## Introduction

C_4_ photosynthesis is used by only 5% of all terrestrial plants, yet these species yield up to a quarter of the Earth’s primary productivity (Jones, 2011; Sage and Zhu, 2011; Sage *et al.*, 2012). The enhanced photosynthetic productivity of C_4_ plants depends on specialized leaf anatomy that compartmentalizes biochemical modifications of the more basic C_3_ pathway (Hatch 1987; Berry et al., 2011, 2013; Garner *et al.*, 2016). The leaves of most C_4_ plants separate two sets of photosynthetic reactions into different leaf cell types, called Kranz anatomy, that consist of an outer mesophyll (M) cell layer surrounding an internal ring of bundle sheath (BS) cells, which in turn surround the leaf veins. An exception occurs in ‘single-cell C_4_’ species of the family *Chenopodiaceae* that compartmentalize these same reaction sets into two regions of leaf chlorenchyma cells (Edwards and Voznesenskaya, 2011; Koteyeva *et al.*, 2016). In Kranz species, the initial carboxylation of phosphoenolpyruvate (PEP) occurs only in the M cells, where phosphoenolpyruvate carboxylase (PEPCase) is specifically localized. CO_2_ incorporation into the Calvin–Benson cycle occurs within chloroplasts of internal BS cells, where Rubisco is specifically located. These C_4_ specializations essentially eliminate metabolically wasteful photorespiration and increase photosynthetic efficiency (Ghannoum *et al.*, 2011; Gowik *et al.*, 2011; Sage *et al.*, 2012; Berry *et al.*, 2013, 2016; Garner *et al.*, 2016).

C_4_ photosynthesis originated ~35 million years ago, evolving independently >60 times across many higher plant families, including both monocots and dicots (Osborne and Freckleton, 2009; Westhoff and Gowik, 2010; Sage et al., 2011, 2012, 2013; Khoshravesh *et al.*, 2016). The occurrence of C_3_–C_4_ intermediates in several present-day genera provides evidence for distinct stages during the evolutionary transition from C_3_ to full C_4_ photosynthesis (Westhoff and Gowik, 2010; Sage *et al.*, 2012; Heckmann *et al.*, 2013; Aubry *et al.* 2014; Khoshravesh *et al.*, 2016; Schulze *et al*., 2016). An initial step is thought to have been the confinement of mitochondrial glycine decarboxylase (GDC) to internal BS cells in leaves of an ancient C_3_ plant (Sage *et al.*, 2013; Schulze *et al.*, 2013; Mallman *et al.*, 2014; Khoshravesh *et al.*, 2016; Schulze *et al*., 2016). Confining the GDC activity to BS cells would establish a photorespiratory pump and increase CO_2_ concentrations around Rubisco, a process also referred to as C_2_ photosynthesis. It is hypothesized that several additional transitional steps followed establishment of the foundational C_2_ state, ultimately leading to present-day full C_4_ pathways (Gowik *et al.*, 2011; Schulze *et al.*, 2013; Khoshravesh *et al.*, 2016; Lin *et al.*, 2016; Schulze *et al*., 2016). Proposed stages include redistribution of mitochondria within BS cells, the evolution of ‘proto-Kranz’ anatomy, and final optimization/activation of the C_4_ cycle. According to this model, the final optimization stage included establishment of BS cell-specific expression of several metabolic genes, some of which already showed BS cell-preferential expression within C_3_ leaves (Aubry *et al.*, 2014). Another proposed process was eliminating the expression of genes encoding Rubisco and other Calvin–Benson cycle enzymes from M cells, leading to the cell type specificity pattern characteristic of present-day C_4_ species.

Rubisco provides an excellent model system to investigate how BS cell specificity for nuclear and plastid-encoded photosynthetic genes might have occurred during C_4_ evolution (Berry *et al.*, 2016). Rubisco is composed of eight large subunits (LSUs) encoded by the chloroplast *rbc*L gene and eight small subunits (SSUs) encoded by a nuclear *Rbc*S gene family (Andersson, 2008; Andersson and Backlund, 2008). Although synthesized in different cellular compartments, anterograde and retrograde signaling processes ensure proportionate amounts of each subunit for the L_8_S_8_ holoenzyme (Patel and Berry, 2008; Berry et al., 2013, 2016). In both C_3_ and C_4_ plants, *rbc*L and *Rbc*S genes are highly regulated in response to external and internal factors (Patel and Berry, 2008; Berry et al., 2013, 2016). External determinants include light, temperature, disease, water, and nutrient availability (Berry et al., 2011, 2013, 2016). Internal factors include developmental stage, cell type, tissue type, and senescence. While transcriptional control of Rubisco gene expression has been implicated in many of these processes, post-transcriptional mechanisms also represent prominent regulatory steps (Patel and Berry, 2008; Berry *et al.*, 2013, 2016).

Post-transcriptional regulation of gene expression is mediated by *cis*-acting regulatory sequences on an mRNA, usually within 5' or 3' untranslated regions (UTRs) (Raynaud *et al.*, 2007; Barkan, 2011; Brown *et al.*, 2011; Berry *et al.*, 2013). These are recognized by RNA-binding proteins that regulate mRNA translation, processing, or stability (Raynaud *et al.*, 2007; Tillich *et al.*, 2010; Barkan, 2011; Berry *et al.*, 2013; Bowman *et al.*, 2013). Many post-transcriptional regulation studies in plants have focused on plastid-encoded genes, where such processes depend on nuclear-encoded plastid-targeted RNA-binding proteins (Tillich *et al.*, 2010; Barkan, 2011; Berry *et al.*, 2013; Bowman *et al.*, 2013). Several classes of binding proteins specifically regulate ~100 different chloroplast-encoded mRNAs (Raynaud *et al.*, 2007; Tillich *et al.*, 2010; Barkan, 2011; Berry *et al.*, 2013; Bowman *et al.*, 2013).

The nuclear-encoded *rbc*L RNA S1 binding domain protein (RLSB) is highly conserved across all groups of land plants (Bowman *et al.*, 2013; Yerramsetty *et al.*, 2016). Biochemical, genetic, and evolutionary studies implicate RLSB as a positive post-transcriptional determinant that binds *rbc*L mRNA, thereby affecting its stability and/or translation (Bowman *et al.*, 2013; Berry *et al.*, 2016). In the C_3_ plant Arabidopsis, RLSB accumulates within the chloroplasts of all leaf chlorenchyma cells. In Kranz-type C_4_ species, RLSB accumulates only within Rubisco-containing chloroplasts of internal BS cells (Bowman *et al.*, 2013), and in the single-cell C_4_ plant *Bienertia sinuspersici* only within internal Rubisco-containing central compartment chloroplasts (Rosnow *et al.*, 2014).

As a ubiquitous and highly conserved mRNA-binding protein associated with post-transcriptional *rbc*L regulation, RLSB could play a role in many processes affecting Rubisco production and localization across a wide range of species. This current study extends our previous findings of RLSB localization and function by revealing the stepwise evolutionary progression to full C_4_ in the genus *Flaveri*a (*Asteraceae*), which contains species possessing a range of photosynthetic types (McKown *et al.*, 2005; Sage *et al.*, 2013; Mallman *et al.*, 2014; Lyu *et al.*, 2015). Findings presented here support a model in which evolutionary modification of RLSB production from an ancestral ‘default’ state in C_3_ plants to full BS cell specificity in C_4_ plants contributed to the subsequent cell-specific expression of chloroplast-encoded *rbc*L expression and Rubisco localization, a process most probably initiated at the C_3_–C_4_/C_2_ intermediate stage and completed during the final ‘activation/optimization’ stage of C_4_ evolution. We also show the tight relationship between RLSB and Rubisco localization in photosynthetic tissues, and post-transcriptional control of both RLSB and Rubisco in both C_3_ and C_4_ species. We conclude that the evolutionary acquisition of specialized C_4_ patterning did not modify the most basic ‘default’ aspects of RLSB/Rubisco localization or production, such as accumulation only in green tissues or light regulation, that are probably shared among all plants.

## Materials and methods

### Comparison and phylogeny of RLSB sequences within the genus *Flaveria*

Translated RLSB ortholog sequences from multiple *Flaveria* species (used with permission from Dr. Julian Hibberd, Department of Plant Sciences, University at Cambridge) were aligned using the MUSCLE multiple sequence algorithm (Edgar, 2004) implemented using CLC Main Workbench 7.7.2. and CLC Genomics Workbench 8.0.3 (https://www.qiagenbioinformatics.com/). Translated RLSB sequences from *Arabidopsis thaliana*, *Zea mays*, and *Amborella trichopoda* (Yerramsetty *et al.*, 2016) were included in the alignment as references (Supplementary Fig. S1 at *JXB* online).

For the phylogeny, data from a total of 63 RNA-Seq libraries of 16 *Flaveria* species were obtained from the National Center for Biotechnology Information (NCBI) sequence read archive (SRA) database (Supplementary Table S1). Initial removal of low quality reads and adaptor trimming was performed with Trimmomatic (Bolger *et al.*, 2014). Filtered reads were assembled with SOAPdenovo-Trans (version 1.04) with eight different K-mers (25, 35, 45, 55, 65, 75, 85, and 95)(Xie *et al.*, 2014). The assembled contigs were merged and redundancy removed using Evidential gene (http://arthropods.eugenes.org/EvidentialGene/evigene/). The number of contigs, total length of transcripts, N50 length, and BUSCO2 quality assessment (Simão *et al.*, 2015) were reported for each species (Supplementary Table S2). The Arabidopsis RLSB gene (AT1G71720) was mapped against the individually assembled contigs using TBLASTN from BLAST (Camacho *et al.*, 2009), and only full-length RLSB sequence were retained. For fragmented RLSB sequence from low quality assembled transcripts species, filtered RNA-Seq reads were mapped to fragmented RLSB sequence by Bowtie2 with the local alignment option (Langmead and Salzberg, 2012). The mapped reads were converted to fasta and quality file, and assembled with fragmented RLSB sequence using Phrap (http://phrap.org/). The contigs from this strategy were examined for full-length coverage by ORFfinder (Wheeler *et al.*, 2003) and SmartBLAST (https://blast.ncbi.nlm.nih.gov/smartblast/). A set of 16 *Flaveria* RLSB genes and three strictly defined outgroup RLSB genes from *Asteraceae* were used to reconstruct phylogeny. The RLSB transcript sequences were converted to coding sequences (CDS) and peptide sequences using TransDecoder (Haas *et al.*, 2013). The peptide sequence from this result were aligned using MUSCLE (Edgar, 2004), and corresponding codon sequence were aligned on peptide alignment by PAL2NAL (Suyama *et al.*, 2006). The alignments were filtered if the position has >50% of gap or the length of the alignment block is smaller than 5 bp by Gblocks (Castresana, 2000). The RLSB tree was constructed using the maximum likehood (ML) approach with the general time-reversible (GTR) substitution+Γ (gamma) model and 1000 bootstrap replicates by RAxML (Stamatakis, 2014). The proper substitution model was selected by PartitionFinder 2 (Lanfear *et al.*, 2017) among the GTR, GTR+Γ, and GTR+Γ+Ι (inverse). The tree was rooted to outgroup species (Supplementary Table S3) RLSB genes which were downloaded from BLAST4OneKP (Matasci *et al.*, 2014)

### Plant material, growth conditions, and tissue sampling

Seed for *F. pringlei* (C_3_), *F. robusta*, *F. linearis* (C_3_–C_4_), *F. palmeri* (C_4_-like), and *F. bidentis* (C_4_) were obtained from Dr Rowan Sage, University of Toronto. For standard growth conditions, seeds were germinated and plants were grown in a greenhouse using artificial soil with a 14 h d^–1^ cycle under 170–200 µmol photons m^−2^ s^−1^. Leaf immunolocalizations used regions midway between the apex and base of young fully expanded 4 cm long leaves, collected from the third node below the apical meristem. Leaf, stem, flowers, and root tissues for immunoblot analysis were collected from 10-week-old plants. Stem samples were taken 10 cm down from the apical meristem. Whole flowers (all four whorls) were used since the small size and compactness of *Flaveria* flowers made it difficult to separate individual flower parts. Root samples consisted of both primary and lateral clippings.

For light regulation studies, etiolated ‘dark-grown’ *F. pringlei* (C_3_) and *F. bidentis* (C_4_) were germinated and grown for 10–14 d in light-proof containers within a dark room (Berry *et al.*, 1988, 1990). Hypocotyls with both cotyledons (stem cut with a scalpel just below the cotyledons) were harvested from etiolated plants under a dim green safelight and immediately placed into a ground glass tissue homogenizer on ice with appropriate buffers for protein or RNA extraction (Bowman *et al.*, 2013). In parallel, hypocotyls/cotyledons were harvested from light-grown *Flaveria* germinated and grown for 10–14 d in a growth chamber with standard lighting. For plants transferred from dark to light (‘greening’), etiolated *Flaveria* seedlings grown in darkness as described above for 8–12 d were transferred into the illuminated growth chamber for 48 h, after which the greening hypocotyls/cotyledons were harvested and frozen.

### Immunolocalization

Serial sectioning of leaf mid-regions from *Flaveria* species described above were prepared for immunolocalization as described (Bowman *et al.*, 2013). The sections were reacted with RLSB, Rubisco LSU, or PEPCase primary antisera at 4 °C overnight (Berry *et al.*, 1988; Bowman *et al.*, 2013), and then reacted with secondary goat anti-rabbit antisera conjugated to Alexa Fluor^®^ 546 (Life Technologies) for 1 h at room temperature. Visualization and image analysis was performed with an LSM710-InTune Confocal Microscope System using the ×20 objective. A 529 nm laser was used for excitation of Alexa Fluor^®^ 546, and emission was collected at 564–577 nm. Images were processed and analyzed using Zen Imaging software (Carl Zeiss).

### Protein extraction and analysis

Total protein extracts were prepared from *Flaveria* leaves, hypocotyls, flowers, roots, and stems as described (Bowman *et al.*, 2013). Equal amounts of total protein were loaded into lanes of an SDS–polyacrylamide gel for analysis by immunoblotting. Gels were electroblotted to either polyvinylidene fluoride (PVDF) (Bio-Rad) or nitrocellulose membranes (GE Healthcare), and then reacted with antisera for RLSB, Rubisco LSU, or PEPCase (Bowman *et al.*, 2013). Images were acquired and analyzed using a Bio-Rad Gel Doc™ XR+ System with Image Lab™ Image Capture and Analysis Software.

*In vivo* protein synthesis in light-grown, etiolated, or 48 h light-transferred (greening) *F. pringlei* (C_3_) and *F. bidentis* (C_4_) was performed as described previously (Berry *et al.*, 1985, 1990). Briefly, freshly cut hypocotyls (immersed in water and cut with a scalpel midway between the root and cotyledons) were placed into 500 μl of labeling solution consisting of 100 μCi of [^35^S]methionine/cysteine Express Labeling Mix (PerkinElmer NEN Radiochemicals) and 400 μl of water. After 1 h incubation either in complete darkness or under standard lighting conditions, labeled protein extracts were prepared from hypocotyls with both cotyledons (with the stem cut off just below the cotyledons after labeling) using equal wet weight of material, cleared in a microfuge to remove insolubles, and stored at –20 °C. Equal amounts of labeled total protein extracts from both species and all three illumination conditions were immunoprecipitated by incubating with RLSB, LSU, and PEPCase antisera overnight with rotational mixing at 4 °C, and antigen–antibody complexes were precipitated using Pansorbin Staph A cells (Millipore) as described (Berry *et al.*, 1985, 1990; Bowman *et al.*, 2013). The immunoprecipitates were separated by SDS–PAGE and the labeled proteins were visualized using a Storm^®^ phosphorimager with ImageQuant software version 4 (GE Healthcare).

### RNA isolation and real-time quantitative PCR

RNA was isolated from hypocotyls of light- and dark-grown (etiolated) *F. pringlei* and *F. bidentis* using an RNeasy Plant Mini Kit (Qiagen) according to the manufacturer’s protocol. cDNA synthesis was performed using an iScript^®^ cDNA synthesis kit (Bio-Rad) with oligo(dT) and random primers included with the kit. Quantitative reverse transcription–PCR (qRT–PCR) was performed with SYBR Green Supermix (Bio-Rad) on a MyiQ™2 Two Color Real-Time PCR Detection System (Bio-Rad), using primers specific for each transcript analyzed (Supplementary Table S4), as indicated in the figures. For each sample, reactions were normalized to actin mRNA using 2^−ΔΔCt^ calculations, and statistical significance was calculated using Student’s *t*-test. For each bar shown in the graphs, *P*-values were <0.05. All data shown represent at least two technical repeats of three independent experiments.

## Results

### RLSB orthologs in *Flaveria* species

RLSB orthologs were identified within available transcriptomes from 16 species of the dicot genus *Flaveria* showing C_3_, C_3_–C_4_ intermediate, C_4_-like, or fully C_4_ characteristics. The predicted RLSB protein sequences used for alignment (Supplementary Fig. S1) were all highly conserved along their entire lengths (>70% overall identity), with the most conserved regions (>90%) occurring within the S1 binding domain (red highlight in Supplementary Fig. S1). Gaps at the N-terminal portion of the alignments occur primarily within the plastid transit sequence, so that the size of the proteins showed some variation between species (ranging from 475 to 502 amino acids). Their strong overall similarity with homologs characterized in Arabidopsis and maize suggests they may be associated with the same *rbc*L regulatory activity described for those plant species (Bowman *et al.*, 2013). These findings are consistent with a previous study that identified strongly conserved RLSB homologs within such diverse plant species as the basal angiosperm *A. trichopoda*, the C_3_ dicot Arabidopsis, and the C_4_ monocot *Z. mays*, even extending as far as Charophyte algae that are considered to be most closely related to the common ancestor of all land plants (Supplementary Fig. S1; Yerramsetty *et al.*, 2016). Previous findings have shown that as with most eudicots, RLSB occurs as a single-copy gene in *Flaveria* species (Yerramsetty *et al.*, 2016).

**Fig. 1.**
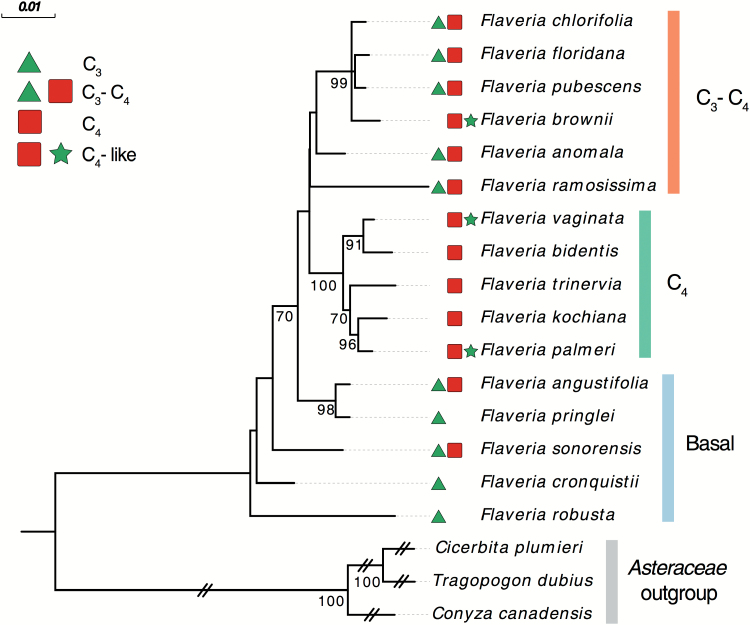
Phylogeny of RLSB orthologs of 16 *Flaveria* (C_3_, C_3_–C_4_/C_2_, C_4_-like, and C_4_) and three outgroup species in *Asteraceae*. A phylogenetic tree was constructed from protein and coding sequences using maximum likelihood (RAxML) to determine the distance. The tree was rooted to outgroup species belonging to *Asteraceae*. Numbers on internal nodes indicate support values with 1000 bootstrap samples, and bootstrap values ≥70 are shown. Branch lengths indicate the number of nucleotide substitutions, and the scale bar represents 0.01 substitutions. The leaf labels on the left along with the species name show photosynthesis status. The main groups are defined on the right according to their clade. (This figure is available in colour at *JXB* online.)

A phylogenetic tree based on available RLSB sequences from 16 *Flaveria* species shows that divergence between homologs, while very low, does show some correlation with C_3_ to C_4_ evolution within this genus (Fig. 1). Based on this phylogenic tree, RLSB sequences of basal species *F. robusta* and *F. cronquistii*, *F. pringlei* representing the ancestral C_3_ state of this genus (McKown *et al.*, 2005; Kümpers *et al.*, 2017), and the C_3_-C_4_*F. angustifolia* and *F. sonorensis*, representing the first step toward C_4_, are more similar to each other than they are to RLSB sequences of the C_3_–C_4_ species (*F. chlorifolia*, *F. floridana*, *F. pubescens*, *F. anomala*, and *F. ramosissima*). Similar to other studies, the C_4_-like species *F. brownii* is closer to the C_3_–C_4_ like group, and does not group with other C_4_-like or C_4_ species. This finding is consistent with previous predictions that there were at least two independent evolutionary events towards C_4_-like and C_4_ photosynthesis within the genus *Flaveria* (Powell, 1978; McKown *et al.*, 2005), and suggests the possibility that RLSB might have played a role in these events. RLSB homologs of the later stage C_4_-like *F. palmeri* or *F. vaginata*, and the fully C_4_*F. bidentis*, *F. kochiana*, and *F. trinervia*, show increasingly more divergence from the earlier species. The fact that C_4_-like *F. vaginata* and *F. palmeri* are closer to completely C_4_*F. bidentis* and *F. kochiana*, respectively, suggests the presence of more than one evolutionary event that led to the establishment of complete ‘C_4_-ness’ in this genus. The short branch lengths of C_4_-like species *F. vaginata* and *F. palmeri* may also suggest reversal events which led to establishment of a C_4_-like state from the advanced C_4_ state, although this cannot be said with certainty based on just this level of analysis. While this phylogeny based solely on RLSB sequences shows some variation from other *Flaveria* phylogenies (Kopriva *et al.*, 1996; McKown *et al.*, 2005; Mallman *et al.*, 2014; Lyu *et al.*, 2015), the overall trend indicates that small alterations in RLSB sequence accompanied the progression from ancestral C_3_ towards fully C_4_ photosynthesis within the genus *Flaveria*. It is possible that such variations in sequence, although not pronounced, could reflect functional or accumulation differences of RLSB orthologs among the different photosynthetic states.

### RLSB co-localizes with Rubisco LSU across the C_3_ to C_4_ species evolutionary gradient

As a contributing factor in the post-transcriptional regulation of *rbc*L expression, RLSB could have been important for establishing BS cell-specific Rubisco localization during C_4_ evolution. The genus *Flaveria* provides an excellent model system to test this hypothesis, due to the presence of multiple photosynthetic states associated with the evolutionary transition (McKown and Dengler, 2007, Mallman *et al.*, 2014; Lyu *et al.*, 2015). For this study, immunolocalization was used to characterize RLSB and Rubisco LSU accumulation patterns in the following representative species of *Flaveria*: *F. robusta* and *F. pringlei*, representing C_3_ type, *F. linearis* representing C_3_–C_4_/C_2_ intermediates, *F. palmeri* with C_4_-like traits, and *F. bidentis* representing a fully C_4_ species. These species were chosen for this study based on their photosynthetic type, distinctive leaf morphologies, their availability, and being highly amenable to leaf sectioning and antibody labeling.

**Fig. 2.**
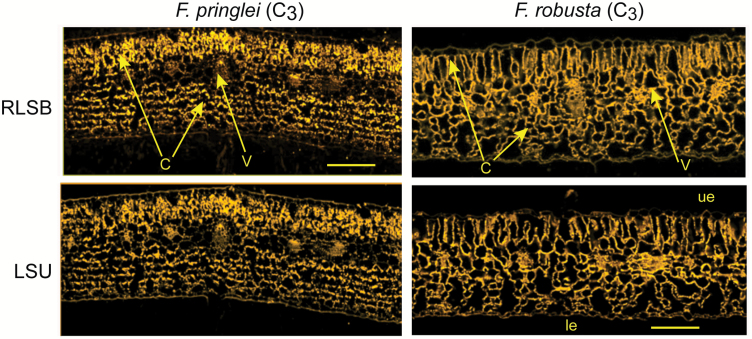
Immunolocalization of RLSB and Rubisco LSU in leaves of C_3_*Flaveria* species. Confocal images of *F. pringlei* (left column) and *F. robusta* (right column) adjacent serial cross-sections taken from a region midway between the leaf apex and base. Sections were incubated with the indicated primary antiserum, and then reacted with Alexafluor 546-conjugated secondary antibody. Images were captured using the ×20 objective of a LSM 710 ‘in tune’ confocal microscope. Images show localization of RLSB (top panels) and Rubisco LSU (bottom panels). Note that these images show typical C_3_ leaf anatomy, with both photosynthetic proteins distributed throughout all of the leaf chlorenchyma cells. Significant anatomical features identified in the leaf cross-sections are labeled as follows: V, vascular bundles; ue, upper epidermis, le, lower epidermis; C, chlorenchyma cells that harbor chloroplasts. Scale bar=150 µm. (This figure is available in colour at *JXB* online.)

In the C_3_ species *F. robusta* and *F. pringlei*, RLSB and LSU were co-localized and equally distributed within all of the leaf chlorenchyma cells, occurring within the large number of chloroplasts within these cells (Fig. 2). Reminiscent of RLSB and LSU accumulation in the C_3_ dicot Arabidopsis (Bowman *et al.*, 2013), there was no cell type specificity observed among the photosynthetic leaf cells of either plant. The leaves of these species showed typical C_3_ anatomy with no Kranz-like features.

**Fig. 3.**
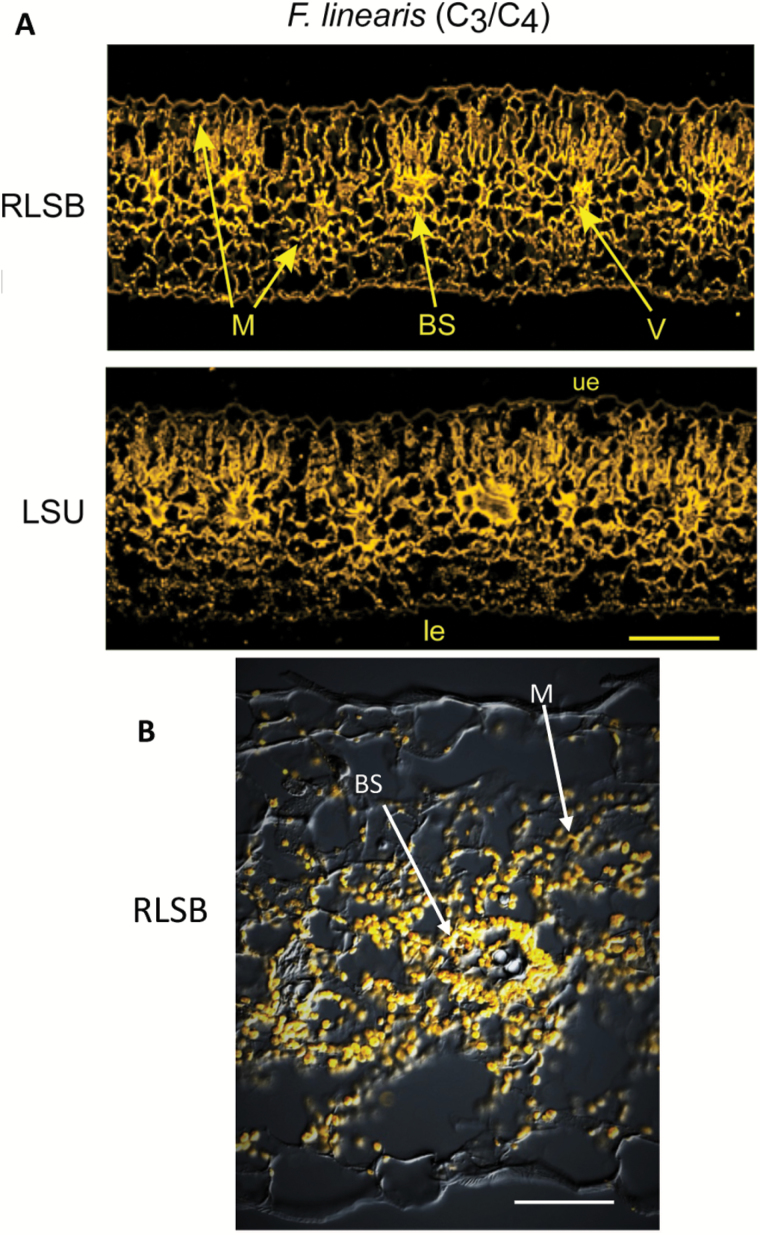
Immunolocalization of RLSB and Rubisco LSU in leaves of a C_3_–C_4_ intermediate *Flaveria* species. Confocal imaging of *F. linearis* leaf adjacent serial cross-sections taken from a region midway between the leaf apex and base. (A) Images in the top two panels were prepared and imaged as described for Fig. 2. Note that these leaves show some C_4_-like features, including larger BS cells with centripetal localization of chloroplasts. RLSB and LSU show some enhancement in the BS chloroplasts, relative to chlorenchyma cell chloroplasts. (B) A combined DIC and RLSB immunolocalization image digitally processed to a higher magnification to show more clearly the proto-Kranz leaf anatomy correlated with enhanced RLSB signal in BS cells of *F. linearis* leaves. Significant anatomical features identified in the leaf cross-sections are labeled. V, vascular bundles; ue, upper epidermis; le, lower epidermis; BS, bundle sheath cells; M, mesophyll (or chlorenchyma) cells. Scale bar for (A)=150 µm, (B)=50 µm. (This figure is available in colour at *JXB* online.)

In the C_3_–C_4_ intermediate *F. linearis*, leaf cross-sections revealed rudimentary ‘proto’-Kranz-like anatomical features, with discernible BS cells surrounding the veins (Fig. 3). Within these BS cells (Fig. 3), the centripetal localization of RLSB- and LSU-containing chloroplasts was evident (Fig. 3). There was some preferential accumulation of these proteins within the BS cells, relative to the surrounding M (chlorenchyma) cells, based on the stronger fluorescence signal detected within the BS cells. The cell-type-preferential compartmentalization of the RLSB and LSU proteins in the leaves of this intermediate species convergent with proto-C_4_-like anatomy appears to represent one step towards the evolution of C_4_ from C_3_ Rubisco production. Thus in leaves with partial C_4_ photosynthesis, there is evidence for the evolutionary beginnings of preferential RLSB and LSU expression within the internal BS cells in leaves of this C_3_–C_4_ intermediate plant.

**Fig. 4.**
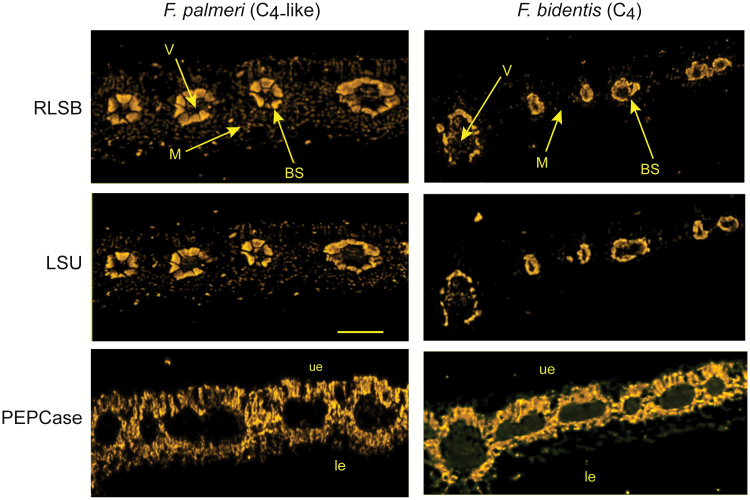
Immunolocalization of RLSB and Rubisco LSU in leaves of C_4_-like and fully C_4_*Flaveria* species. Confocal imaging of *F. palmeri* (C_4_-like) and *F. bidentis* (C_4_, right column) leaf adjacent serial cross-sections taken from a region midway between the leaf apex and base. Samples were prepared and imaged as described for Fig. 2. Images show localization of RLSB (top panels), LSU (middle panels), and as an M cell-specific control, PEPCase (bottom panels). Note that the *F. palmeri* leaf images show clearly discernible C_4_ leaf anatomy, with large BS cells with centripetal localization of chloroplasts surrounded by a ring of M cells. LSU and RLSB show localization primarily in the BS chloroplasts, with low levels of both proteins observable in M cell chloroplasts as well. In comparison, leaves of the fully C_4_*F. bidentis* show clearly discernible C_4_ leaf anatomy, with large BS cells with centripetal localization of chloroplasts surrounded by a ring of M cells. RLSB and LSU are specifically located to BS chloroplasts. In both photosynthetic leaf types, PEPCase was specifically localized to the M cell cytoplasm. Significant anatomical features identified in the leaf cross-sections are labeled in (B). V, vascular bundles; ue, upper epidermis; le, lower epidermis; BS, bundle sheath cells; M, mesophyll cells. Scale bar=150 µm. (This figure is available in colour at *JXB* online.)

A side-by-side comparison of leaf sections from the C_4_-like *F. palmeri* and the fully C_4_*F. bidentis* showed differentiation between BS and M cells in both species to be significantly more pronounced than in the C_3_ and C_3_–C_4_ intermediate (Fig. 4). Leaf BS cells in both of these advanced C_4_ types were much larger than in the C_3_ and C_3_–C_4_ intermediates, with RLSB and LSU proteins predominantly located in the BS cells. In leaves of the C_4_-like *F. palmeri*, there was still some signal of the two proteins in M cells, but at reduced levels relative to BS cells (Fig. 4, top left panel). These observations suggest that in *F. palmeri*, which has progressed beyond the midpoint towards C_4_ evolution, most (but not all) of the Rubisco has become compartmentalized to the inner ring of morphologically distinct BS cells. RLSB localization also followed this pattern; similar to LSU, most of the RLSB protein accumulation was observed in BS cells, with low but still easily detectable levels occurring in the M cells (Fig. 4, left middle panel). Consistent with previous findings (Bowman *et al.*, 2013), leaves of the fully C_4_*F. bidentis* showed both LSU and RLSB proteins exclusively localized within chloroplasts of the fully differentiated leaf BS cells, with little or no accumulation within M cells of this species (Fig. 4, right top and middle panels).

For a cell specificity comparison, in both C_4_-like *F. palmeri* and the fully C_4_*F. bidentis*, PEPCase protein accumulation was highly specific to the cytoplasm of M cells, and not BS cells, in the characteristic C_4_ pattern. Thus, cell specificity for PEPCase was observed in corresponding leaf sections of both photosynthetic types, while RLSB and Rubisco specificity was observed only in the fully C_4_*F. bidentis*. It is likely that processes responsible for the establishment of complete M cell specificity occur independently and at an earlier stage than those responsible for BS cell specificity along the C_3_ to C_4_ evolutionary progression.

### RLSB and LSU accumulation in photosynthetic and non-photosynthetic tissues of C_3_ and C_4_*Flaveria* species

**Fig. 5.**
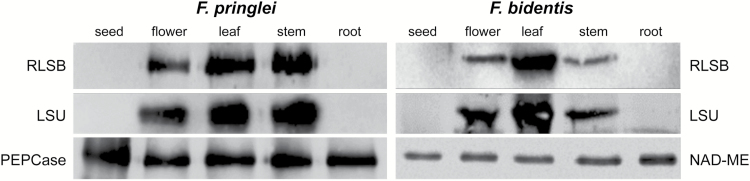
Abundance of RLSB and Rubisco LSU proteins in photosynthetic and non-photosynthetic tissues of *F. pringlei* (C_3_) and *F. bidentis* (C_4_). For both *Flaveria* species, total protein extracts were prepared from each of the tissues indicated. Equal amounts of total protein were loaded into each lane, separated by SDS–PAGE, and transferred to nitrocellulose for immunoblotting using the antisera indicated. The left panel shows RLSB, LSU, and PEPcase (the non-photosynthetic C_3_ form) in seeds, flowers, leaves, stems, and roots of *F. pringlei*, as indicated. The right panel shows RLSB, LSU, and NAD-ME (a non-photosynthetic enzyme in NADP-ME species) in seeds, flowers, leaves, stems, and roots of *F. bidentis*. Note that, due to difficulty in separation of individual flower sections, the flower extracts contained green calyx tissue. For the bottom PEPCase immunoblot of *F. pringlei* tissues, all of the lanes were loaded with excess (10-fold) of the protein extracts compared with the RLSB and LSU immunodetection gels, in order to better detect the very low amounts of this protein present in the C_3_ species.

As a potential positive regulator of *rbc*L expression, RLSB would be expected to show the same tissue-specific patterns of accumulation as the protein it regulates, the Rubisco LSU, throughout different photosynthetic and non-photosynthetic plant tissues. To determine if RLSB and Rubisco accumulation correlate in tissues other than leaves, and if tissue specificity patterns are conserved among plant species using the different photosynthetic pathways, immunoblot analysis was performed using protein extracts from seeds, flowers, leaves, stems, and roots of C_3_*F. pringlei* and C_4_*F. bidentis* (Fig. 5). PEPCase, used as a loading control for the *F. pringlei* tissues, is a low abundance non-photosynthetic protein in leaves and other tissues of C_3_ species (Berry *et al.*, 2011, 2013). To better visualize this protein, the gel used for the PEPCase immunoblot shown in Fig. 5 was loaded with a higher concentration (10-fold higher) of the equalized protein extracts than used for the RLSB and LSU gels. It should be noted that while at least some C_3_ and all C_4_ plants contain different forms of PEPCase (Berry *et al.*, 2011; Ruckle *et al.*, 2007; Burgess *et al.*, 2016), the polyclonal antiserum used here was prepared against full-length protein purified from amaranth leaves (Wang *et al.*, 1992) and would not distinguish between different forms in western analysis. For C_4_*F. bidentis*, the NAD-dependent malic enzyme (NAD-ME), a non-photosynthetic protein in this C_4_ species, was used as a loading control.

The tissue-specific accumulation patterns for RLSB and LSU did not vary between C_3_ and C_4_ species. Both proteins were most abundant in leaves and in green stems, both of which are photosynthetic tissues (Fig. 5). These proteins were also detected in flowers, most probably due to the presence in these extracts of green tissue from the calyx. No RLSB protein was detected in the non-photosynthetic seeds and roots, where LSU was also absent. In contrast, the non-photosynthetic PEPCase and NAD-ME proteins were found at approximately equal levels in each tissue type of *F. pringlei* and *F. bidentis*, respectively.

The close correlation between RLSB and LSU accumulation in these different plant tissues provides further support for RLSB as a key determinant of LSU production and localization. Modified accumulation patterns for the regulatory protein RLSB between C_3_ and C_4_ plants, like Rubisco, occurred only in leaves (from Figs 2–4). Thus, the co-ordinated C_3_ to C_4_ evolutionary progression of RLSB and LSU cell type expression patterns in the direction of enhanced ‘C_4_-ness’ was an occurrence specific to leaf cells that did not affect the accumulation of these proteins in any tissues other than leaves.

### Light regulation of RLSB in C_3_ and C_4_*Flaveria* species

**Fig. 6.**
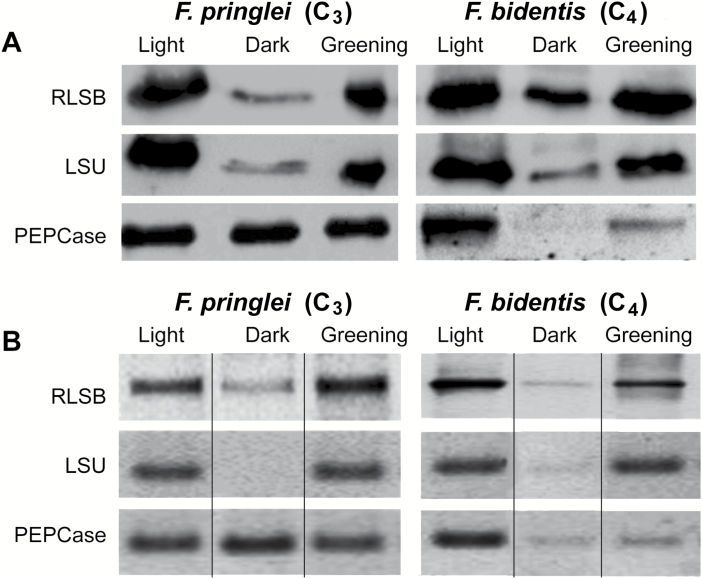
Abundance and *in vivo* synthesis of RLSB and Rubisco LSU proteins in etiolated, light-grown, and 48 h greening *F. pringlei* (C_3_) and *F. bidentis* (C_4_). Proteins were isolated from hypocotyls of *F. pringlei* (C_3_) and *F. bidentis* (C_4_) seedlings grown in light, dark, or 48 h greening conditions. (A) Immunoblot showing relative abundance of RLSB, LSU, and PEPCase under the three different illumination conditions. Equalized amounts of total proteins from hypocotyls of *F. pringlei* (C_3_) and *F. bidentis* (C_4_) grown under the three conditions were loaded into each lane. Total protein extracts were separated by SDS–PAGE, and analyzed by immunoblot using the antisera indicated. Note that the *F. pringlei* PEPCase immunoblot was loaded with excess protein extract as described for Fig. 5. (B) Detached hypocotyls from *F. pringlei* and *F. bidentis* seedlings grown under the different illumination conditions were labeled with [^35^S]methionine/cysteine, and LSU, RLSB, and PEPCase were immunoprecipitated from equal amounts of the labeled extracts. Immunoprecipitation reactions were separated by SDS–PAGE, and visualized and quantitated using a phosphorimager with ImageQuant software. Note that for *F. pringlei*, PEPCase was immunoprecipitated from 10-fold more extract than used for RLSB and LSU. For clarity, the original order of sample loading in (B) was digitally rearranged to correspond to the order of sample loading of (A), as highlighted by vertical lines between the gel lanes. Other than editing of the loading order, there was no change in exposure or other modifications made to the figure. For comparison, the original gel in (B) showing the non-modified order of sample loading is included as Supplementary Fig. S2.

The post-transcriptional regulation of photosynthetic gene expression by light has been well documented and is especially prominent in the regulation of *rbc*L and other plastid-encoded genes (Patel and Berry, 2008; Berry et al., 2011, 2013, 2016). To determine if the nuclear-encoded RLSB *rbc*L mRNA-binding protein itself is regulated by light, and if this regulation has been modified during the transition from C_3_ to C_4_ photosynthesis, the accumulation and synthesis of RBCL and LSU proteins was examined in hypocotyls from light-grown, dark-grown (etiolated), and 48 h ‘greening’ *F. pringlei* (C_3_) and *F. bidentis* (C_4_) plants. Hypocotyls were used for the light regulation experiments because the etiolated *Flaveria* seedlings did not produce leaves. Previous studies from our laboratory demonstrated that these tissues provide an excellent system to study light-mediated gene expression (reviewed in Patel and Berry, 2008; Berry *et al.*, 2011, 2013). For the experiments shown in Fig. 6, seeds of each species were germinated and grown under normal illumination (Light), in complete darkness (Dark), or in darkness and then transferred to light for 48 h (Greening), as described in the Materials and methods. For both *Flaveria* species, we found that 10–14 d growth of seedlings under light and dark conditions was optimal for experimental viability, light responsiveness, and cotyledon development.

For immunoblot analysis (Fig. 6A), equal amounts of total protein from hypocotyls of each species grown under the conditions indicated were loaded and separated by SDS–PAGE, and subjected to immunoblot analysis as described for Fig. 5 (including using higher concentration of extracts to detect PEPCase in C_3_*F. pringlei*). For both the C_3_ and C_4_*Flaveria* species, there was substantially more LSU and RLSB in light-grown seedlings, relative to plants of the same age grown in total darkness. When the etiolated plants were transferred to light for 48 h, levels of both proteins increased to levels observed in seedlings grown under normal illumination conditions. The reduction in RLSB in response to darkness (etiolation) occurred in both species, although to a lesser degree in the C_4_ species relative to the C_3_ species (Fig. 6A). LSU accumulation in these species reflected RLSB accumulation, with significant reductions in the dark-grown seedlings relative to light-grown, and an increase in dark-grown seedlings after 48 h transfer to light (Fig. 6A).

*In vivo* protein synthesis was analyzed by using seedlings of *F. pringlei* (C_3_) and *F. bidentis* (C_4_) that were labeled with [^35^S]methionine/cysteine while growing under the different illumination conditions. Labeled proteins were extracted from the hypocotyls, and LSU, RLSB, or PEPCase were immunoprecipitated from equalized amounts of total labeled protein. In both the C_3_ and C_4_ species, the light-associated changes in RLSB and LSU accumulation were mirrored by corresponding changes in *in vivo* synthesis for each protein. For both species, *in vivo* synthesis of LSU and RLSB was easily detectable in light-grown seedlings (Fig. 6B), while synthesis of both proteins was significantly reduced in the dark-grown seedlings. After transfer of etiolated seedlings to light, levels of LSU and RLSB synthesis increased, reaching normal light-grown levels by 48 h following transfer.

Unlike RLSB and LSU, the amounts and synthesis of non-photosynthetic PEPCase in the C_3_*F. pringlei* hypocotyls was not affected by changes in illumination (Fig. 6A, B, bottom left panels). In contrast, in C_4_*F. bidentis*, accumulation and synthesis of photosynthetic PEPCase were reduced in darkness, relative to light, and increased in response to 48 h of illumination (Fig. 6A, B, bottom right panels). This is in agreement with previous studies showing that the photosynthetic form of this enzyme acquired light regulation during its modification from metabolic to photosynthetic function during C_4_ evolution (Berry *et al.*, 2011, 2013). However, it is important to note that 48 h after transfer to light increased PEPCase and its synthesis had not reached the more abundant levels observed in the hypocotyls of light-grown seedlings. Therefore, while light regulation is a characteristic of all three photosynthetic proteins in C_4_*F. bidentis*, for these tissues and conditions, light-induced synthesis of photosynthetic PEPCase appears to lag behind RLSB and LSU, probably requiring longer period of growth under illumination to achieve normal levels of synthesis and accumulation.

Taken together, it is apparent that at the levels of protein accumulation and synthesis, nuclear-encoded RLSB is similar to the chloroplast-encoded LSU, with production of both proteins being light regulated in C_3_ and C_4_ plants. The shared light regulation of RLSB at opposite ends of the C_3_ to C_4_ evolutionary spectrum differs from PEPCase, a nuclear-encoded protein recruited to a photosynthetic function that shows light-regulated production only in the C_4_ species.

**Fig. 7.**
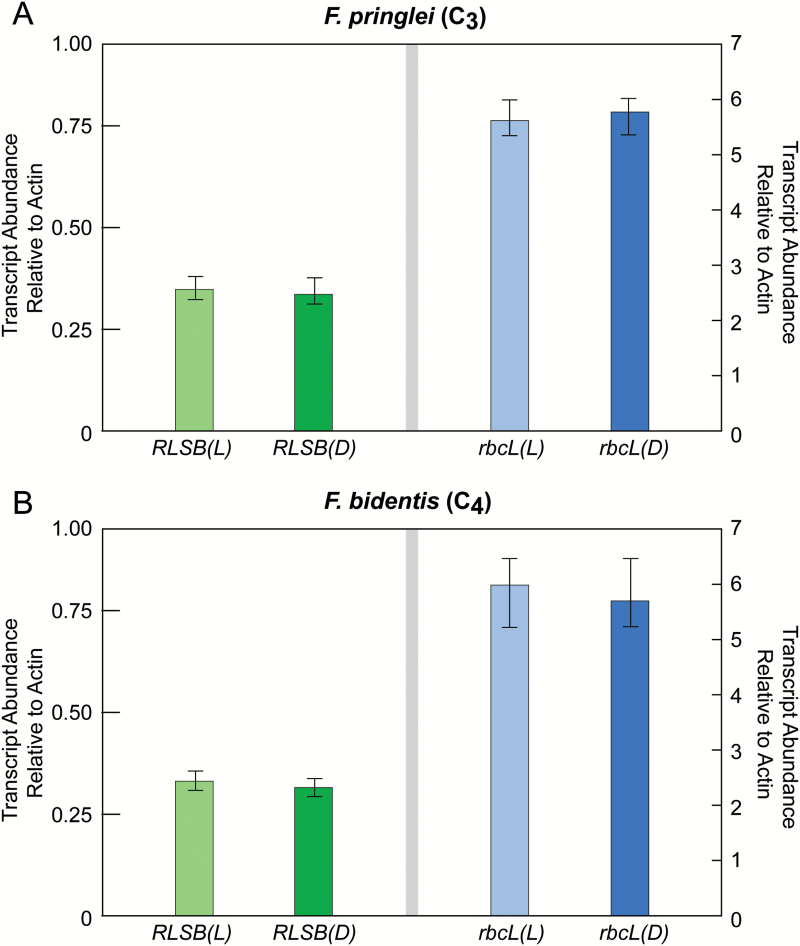
*RLSB* and *rbc*L mRNA in *F. pringlei* (C_3_) and *F. bidentis* (C_4_) in response to light. For each panel, RNA was extracted from hypocotyls of *F. pringlei* (C_3_) and *F. bidentis* (C_4_) seedlings grown in light or complete darkness (etiolated). Transcript levels of both genes were analyzed by qRT–PCR using primers specific for each sequence. Quantification of transcript levels was standardized to actin mRNA. (A) Relative levels of *RLSB* and *rbc*L mRNA accumulation in *F. pringlei* seedlings grown under the conditions indicated. (B) Relative levels of *RLSB* and *rbc*L mRNA in *F. bidentis* seedlings grown under the conditions indicated. Data in both panels represent at least two technical repeat reactions of three independent experiments. Note differences in scale for panels showing *RLSB* and *rbc*L mRNAs, indicating that these two transcripts accumulate to substantially different levels in both plants under each condition. (This figure is available in colour at *JXB* online.)

Analysis of *RLSB* and *rbc*L mRNA using qRT–PCR showed that in both *Flaveria* species, as in the C_3_ dicot Arabidopsis and the C_4_ monocot maize (Bowman *et al.*, 2013), mRNAs encoding RLSB are much less abundant than those encoding *rbc*L (note the difference in *y*-axis scales for *RLSB* and *rbc*L in Fig. 7). In both C_3_*F. pringlei* and C_4_*F. bidentis*, the abundance of *rbc*L and *RLSB* mRNAs was not affected by illumination, with approximately equal levels of each transcript present in both light-grown and dark-grown plants (Fig. 7). This is in clear contrast to the RLSB and LSU proteins, both of which showed significant reductions in accumulation and synthesis in dark-grown seedlings relative to those grown in light (Fig. 6A, B). The lack of correlation between the accumulation of transcripts and their encoded proteins is indicative of regulation at the level of translation, or possibly protein stability. Thus, like many proteins associated with C_3_ and C_4_ photosynthetic processes, including the Rubisco LSU and SSU subunits (Patel and Berry, 2008; Berry *et al.*, 2013, 2016), RLSB expression/accumulation appears to be post-transcriptionally regulated by light.

## Discussion

### RLSB and C_4_ evolution

Plastid-encoded genes are regulated at multiple steps, from transcription, RNA processing, transcript stabilization, and translation (Raynaud *et al.*, 2007; Tillich *et al.*, 2010; Barkan, 2011; Berry *et al.*, 2013). Each of these steps involves multicomponent complexes of interacting proteins, many of which are encoded in the nucleus as participants in anterograde signaling and gene regulation. Our previous findings demonstrated that reduced RLSB leads to corresponding reductions in *rbc*L mRNA (Bowman *et al.*, 2013), providing evidence that this protein in itself is a determinant of transcript stability. RLSB was purified based on its ability to bind the 5' portion of this mRNA (Bowman *et al.*, 2013), where sequences required for stability/degradation of *rbc*L and other plastid transcripts are located (Salvador *et al.*, 2011; Berry *et al.*, 2013). However, RLSB probably does not function on its own. We suspect that other proteins, some cell or tissue specific and others more general, might interact with RLSB as part of a complex to mediate final translation of LSU protein from stabilized *rbc*L mRNA. Reductions in RLSB or any other single component would lead to inactivation/destabilization of the entire complex, causing the observed decreases in *rbc*L mRNA and LSU protein observed in our previous studies. This phenomenon occurs for many interacting proteins, including Rubisco and other chloroplast complexes (Choquet *et al.*, 2003; Cohen *et al.*, 2005, 2006; Duncan and Mata, 2011). During C_4_ evolution, only one key component of such a regulatory complex, such as RLSB, would need to become down-regulated in M cells to achieve BS cell-specific activity. As a conceptual example, the BS cell-specific localization of only one of four GDC subunits, the P subunit, in C_3_–C_4_ intermediates of *Flaveria* and other genera demonstrates how a single component might become the cell specificity determinant for an entire complex (Sage *et al.*, 2012; Schulze *et al.*, 2013; Khoshravesh *et al.*, 2016; Schulze *et al*., 2016). Similarly, reducing the expression of only the H subunit in M cells of transgenic rice correspondingly reduces GDCH activity within those cells (Lin *et al.*, 2016). On the other hand, any potential overproduction of RLSB alone in any tissues or conditions might not have an effect on overall Rubisco production, at least not a complete effect. Increased Rubisco holoenzyme might not occur without co-overexpression with other interacting components, or even the SSU to interact with and stabilize any excess LSU produced.

As a post-transcriptional regulatory component of *rbc*L mRNA metabolism, RLSB would probably be involved with many cell- and tissue-specific aspects of Rubisco gene expression in C_3_ and C_4_ plant species (Bowman *et al.*, 2013; Rosnow *et al.*, 2014; Yerramsetty *et al.*, 2016). This is especially significant for the evolution of C_4_ photosynthesis where nuclear-encoded RLSB, through anterograde regulation, could assist in localizing the expression of the chloroplast-encoded *rbc*L gene and therefore overall Rubisco production to leaf BS cells. According to this model, cell type-specific regulation of plastid genes would be anchored to cell-specific expression of nuclear-encoded regulatory genes, thereby co-ordinating cell specificity between genes encoded within the different cell compartments. Other mechanisms are also likely to be involved in co-ordinating the two compartments, including retrograde signaling and light regulation (Berry *et al.*, 2013; Burgess *et al.*, 2016). This hypothesis is supported by findings presented in the current study, in which RLSB and Rubisco co-localization was observed in several members of the genus *Flaveria* that display different patterns of Rubisco localization depending on the photosynthetic pathway utilized. Co-localization of LSU with its associated transcript-binding protein RLSB occurred within mature leaves across a range of *Flaveria* species representing C_3_, C_3_–C_4_, C_4_-like, and C_4_ photosynthesis types. This tight association across an evolutionary continuum of ‘C_3_ to C_4_-ness’ provides evidence that RLSB localization has laid down the pattern of Rubisco localization in leaves of the different photosynthetic types, gradually leading to the confinement of Rubisco to BS cells in Kranz-type species with full C_4_ development. Under this scenario, the *rbc*L regulatory protein RLSB is proposed to have played a role in the evolutionary transition from C_3_ to C_4_, with its localization essential for the downstream BS cell-specific localization patterns exhibited by Rubisco.

This hypothesis is also consistent with the fact that, like RLSB (Yerramsetty *et al*, 2016), *rbc*L regulatory and coding sequences are for the most part very conserved throughout all higher plants (Manen *et al.* 1994; Salvolainen et al., 2000; Kapralov et al., 2010, 2011; Sharwood *et al.*, 2016). *rbc*L genes in different plant species do show variations that occur primarily within their coding sequences, with amino acid changes affecting holoenzyme assembly, interactions with Rubisco activase, and enzyme kinetics (Kapralov *et al.*, 2010, 2011; Sage *et al.*, 2012; Sharwood *et al.*, 2016). Some of these have been linked with functional adaptations during the evolutionary transition from C_3_ to C_4_ photosynthesis. However, changes to the *rbc*L coding sequence itself would probably occur independently from modifications in C_3_ to C_4_ gene expression patterns. In fact, non-coding regulatory regions of this gene appear to have been highly conserved, at least among dicots (Manen *et al.*, 1994).

A recent study indicated that Arabidopsis RLSB (designated as PRB1 in that study) shows *in vitro* interactions with biotinylated *ycf*1 mRNA (Yang *et al.*, 2016). In our previous study, we suggested that RLSB could interact with and regulate one or more plastid mRNAs, in addition to *rbc*L, that were not included in our *in vitro* or *in vivo* binding analysis (Bowman *et al.*, 2013). Plastid-encoded *ycf*1 is an essential cell viability gene in many plant species (Asakura and Barkan, 2006; Bölter and Soll, 2017), but it does not occur in all plants. For example, the *ycf*1 gene appears to have been evolutionarily deleted from chloroplast genomes of most grasses, including C_4_ maize (Maier *et al.*, 1995; Drescher *et al.*, 2000; Asakura and Barkan, 2006) which has two RLSB paralogs (Yerramsetty *et al.*, 2016). The function of *ycf*1 has not been clearly established in any plant, and a potential role for this protein in C_4_ capability or evolution is not known.

In the two C_3_ species, *F. robusta* and *F. pringlei*, both RLSB and LSU were found within chloroplasts that were distributed throughout all of the leaf chlorenchyma cells, and were not specific to any one photosynthetic cell type (Fig. 2). In the C_3_–C_4_ intermediate *F. linearis*, some C_4_-like anatomical features, such as the presence of morphologically distinguishable M cells and BS cells, were clearly apparent (Fig. 3). In these leaves, proto-Kranz BS cells were located immediately surrounding the vascular bundles. These cells were larger and contained more chloroplasts than the adjacent M cells. The reduction in M cell chloroplast number in this species relative to BS cells (clearly observable in Fig. 3B) is characteristic of this early stage towards increased C_4_-ness (Stata *et al.*, 2014; Khoshravesh *et al.*, 2016; Lin *et al.*, 2016). In addition to the more numerous chloroplasts, BS cells of *F. linearis* leaves showed increased fluorescence signal representing increased amounts of chloroplast-localized RLSB and LSU proteins relative to the M cells, indicating co-ordinated BS-preferential accumulation of both proteins at this intermediate stage of C_4_ evolutionary development (Fig. 3). As the C_4_-ness increases, the M cell-associated reduction in chloroplast density becomes magnified, as observed in the C_4_-like species *F. palmeri* and the fully C_4_ species *F. bidentis* (Fig. 4). In leaves of the C_4_-like *F. palmeri* (Fig. 4, left panels) the formation of well-defined Kranz anatomy was clearly observable, with the majority of RLSB and LSU being localized to the BS chloroplasts. In these leaves, cell specificity for LSU and the *rbc*L regulator RLSB was not complete, with low levels of both proteins still found within the M cell chloroplasts. In *F. bidentis* (Fig. 4, right panels), the species showing the most advanced full C_4_ stage of evolutionary development, Rubisco and RLSB were both highly specific to leaf BS cells, with little if any of either protein observed within the fully differentiated M cells. At this final stage along the C_3_–C_4_ species gradient, specific localization of RLSB and LSU to leaf BS cells is complete. These observed evolutionary changes exhibited by both RLSB and LSU across these different *Flaveria* photosynthetic types provides strong evidence that C_4_ evolution has incorporated cell-specific modifications to genes encoding both proteins, the nuclear-encoded regulatory protein RLSB and the chloroplast gene it regulates, *rbc*L. These modifications may have occurred in co-ordination with modifications to leaf anatomy, since the small anatomical changes in the C_3_–C_4_ intermediate *F. linearis* were accompanied by changes in the localization of these two proteins.

The progression towards C_4_-ness in *Flaveria* species is associated with changes in the CO_2_ compensation point, with values of C_3_–C_4_ species approximately midway between those of C_3_ and fully C_4_ species (Holaday *et al.*, 1984; Edwards and Voznesenskaya, 2011; Sage *et al.*, 2012; Mallmann *et al.*, 2014; Khoshravesh *et al.*, 2016). Reduced photorespiration is another factor associated with the degree of C_4_-ness, and the photorespiration avoidance efficiencies of C_3_–C_4_ intermediates of *Flaveria* also lie in between the true C_3_ and fully C_4_ species (Sage *et al.*, 2012; Schulze *et al.*, 2013; Mallmann *et al.*, 2014; Khoshravesh *et al.*, 2016; Schulze *et al*., 2016). Enhanced photosynthetic efficiency based on a lowered CO_2_ compensation point and reduced photorespiration are directly related to the internalization of Rubisco within C_4_ leaves. In Kranz species, the only way for Rubisco to become BS cell specific is by modification of default Rubisco gene expression in C_3_ plants to BS cell specificity in C_4_ plants. Post-transcriptional regulation of Rubisco gene expression is likely to play a major role in this process, probably mediated by the *rbc*L RNA-binding protein RLSB (Patel and Berry, 2008; Hibberd and Covshoff, 2010; Berry *et al.*, 2013, 2016; Bowman *et al.*, 2013). It is notable that recent translatome data have shown that RLSB is not preferentially expressed in C_3_ Arabidopsis BS cells (Aubry *et al.*, 2014). This is consistent with our findings of a progressive C_3_ to C_4_ evolutionary transition towards BS specificity for this mRNA-binding protein in *Flaveria* species.

While our previous studies (Bowman *et al.*, 2013; Rosnow *et al.*, 2014) demonstrated the co-localization for RLSB and Rubisco in mature leaves of several C_3_ and C_4_ plants, they did not address if there was any correlation in how the localization patterns developed. If these had in fact occurred independently (i.e. with no progression or co-ordination), this would suggest that RLSB was not associated with the progressive evolution of C_4_ Rubisco localization, and that their co-localization in mature C_3_, as well as Kranz-type and single-cell C_4_ leaves might be more circumstantial and possibly unrelated. In fact, the progressive correlation for RLSB and Rubisco localization across the C_3_ to C_4_ spectrum was striking. Changes in localization for both proteins occurred together with the incremental morphological development of Kranz anatomy and correspond to other progressive changes known to be associated with C_4_ evolution (Holaday *et al.*, 1984; Edwards and Voznesenskaya, 2011; Sage *et al.*, 2012; Mallmann *et al.* 2014; Khoshravesh *et al.*, 2016). Considered together, these findings suggest that change in localization of RLSB-binding protein was one prominent factor, integrated and working in unison with other molecular, physiological, and morphological processes, during the evolutionary progression leading from C_3_ to full C_4_ capability.

### RLSB is associated with tissue specificity and light regulation of *rbc*L expression in C_3_ and C_4_*Flaveria*

Tissue-specific RLSB and Rubisco LSU accumulation patterns in the C_3_ and C_4_*Flaveria* species mirror patterns of mRNA accumulation observed for the C_3_ dicot Arabidopsis (Bowman *et al.*, 2013). Rubisco LSU and RLSB were found to accumulate only in photosynthetic tissues, which included leaves, green stems, and flowers with the green calyx. The finding that RLSB, like LSU, was only in these same green tissues, and not in non-green tissues such as roots and seed, provides further evidence for its role as a regulator of photosynthetic activity (Berry *et al.*, 2013; Bowman *et al.*, 2013; Yerramsetty *et al.*, 2016). Although RLSB accumulation is BS cell specific in the fully C_4_ species, it has retained the same conserved pattern of accumulation in the stems, leaves, and flower sepals as the C_3_ species. Whatever regulatory modification was responsible for restricting *RLSB*/*rbc*L expression to BS cells during C_3_ to C_4_ evolution appears to have occurred only in leaves, without affecting overall tissue-specific accumulation patterns shared with other plant species. Consistent with our hypothesis, this finding suggests tissue-specific accumulation patterns of Rubisco accumulation may be defined by patterns of RLSB accumulation in the different plant tissues. Furthermore, regulatory processes responsible for limiting RLSB accumulation to C_4_ BS cells are probably separable from those that limit its expression to photosynthetic tissues in both C_3_ and C_4_ species, and were not affected by the evolutionary transition to the more evolutionarily derived photosynthetic pathway.

LSU and RLSB protein accumulation (Fig. 6A) and *in vivo* synthesis (Fig. 6B) were found to be light dependent in hypocotyls of C_3_ and C_4_*Flaveria* species. This is consistent with previous studies demonstrating light regulation of *rbc*L expression in many plant species (Patel and Berry, 2008; Berry *et al.*, 2013, 2016). As with cell type and tissue type specificity, light-induced changes in LSU accumulation were mirrored by changes in the accumulation and synthesis of RLSB. These results clearly show that the tight association between RLSB and Rubisco production is maintained in different light conditions for both C_3_ and C_4_ photosynthetic types.

As expected from other studies (Kausch *et al.*, 2001; Berry *et al.*, 2011), synthesis and accumulation of non-photosynthetic PEPCase protein in hypocotyls of C_3_*F. pringlei* was not regulated by light, while the PEPCase in C_4_*F. bidentis* did show light regulation. The accumulation of photosynthetic PEPCase is known to be up-regulated by light in C_4_ plants, due primarily to regulation of transcription. However, light-induced expression of photosynthetic PEPCase was delayed in hypocotyls of *F. bidentis*, so that a longer period of greening may be required for induction of this M cell-specific gene, at least in these tissues. Such findings suggest that the acquisition of light-regulated protein production for the nuclear-encoded RLSB in C_3_ and C_4_ plants and nuclear-encoded photosynthetic PEPCase in C_4_ plants were non-synchronous events that occurred independently during the evolutionary progression from C_3_ to C_4_ in *Flaveria*. In contrast to protein production, a recent study indicates that transcription and accumulation of PEPCase mRNA is light regulated in both C_3_ Arabidopsis and C_4_*Gynandropsis gynandra* (Burgess *et al.*, 2016). Different analytical approaches used in this current study versus the previous study, such as chromatin immunoprecipitation and sequencing analysis (ChIP-SEQ) versus western blot and *in vivo* protein synthesis in different species, suggest that transcriptional and post-transcriptional regulatory mechanisms determining final PEPCase levels may not be the same in all plants. Accumulated findings regarding PEPCase regulation in C_3_ and C_4_ plants are consistent with the model that genes encoding different C_4_ photosynthesis proteins are regulated independently, involving both shared and divergent regulatory processes that vary between species (Hibberd and Covshoff, 2010; Berry *et al.*, 2011, 2013; Burgess *et al.*, 2016; Garner *et al.*, 2016; Williams *et al.*, 2016; Kümpers *et al.*, 2017).

Similar to *rbc*L and *Rbc*S in the C_4_ dicot amaranth and other plants (Patel and Berry, 2008; Berry et al., 2013, 2016), differences in the synthesis and accumulation of Rubisco RLSB and LSU proteins in response to light and dark growth conditions did not correlate with levels of their corresponding transcripts for either C_3_*F. pringlei* (Fig. 7A) or C_4_*F. bidentis* (Fig. 7B). Nuclear-encoded *RLSB* mRNAs and plastid-encoded *rbc*L mRNAs were present at nearly identical levels in seedlings grown under normal illumination (when accumulation and synthesis of both proteins occurred) and in complete darkness (when their accumulation and synthesis did not occur). This lack of correlation between protein and transcript accumulation is indicative of post-transcriptional regulation at the level of translation, or possibly protein stability. Such regulation is characteristic of nuclear- as well as plastid-encoded photosynthesis genes in many C_3_ and C_4_ plant species (Patel and Berry, 2008; Berry *et al.*, 2011, 2013). This finding provides further evidence that the RLSB mRNA-binding protein is closely correlated with post-transcriptional *rbc*L expression, possessing regulatory properties characteristic of many other post-transcriptionally regulated photosynthetic genes.

Several studies have shown that Rubisco activity, protein levels, and transcript accumulation are reduced in the leaves of C_3_ relative to C_4_ plants, which contributes to the increased nitrogen-use efficiency of C_4_ species (Patel and Berry, 2008; Gowik and Westhoff, 2011; Sage *et al.*, 2012; Carmo-Silva *et al.*, 2015; Garner *et al.*, 2016; Kümpers *et al.*, 2017). In Figs 6 and 7, similar levels of LSU protein synthesis and accumulation, as well as *rbc*L mRNA levels, were observed in light-grown and greening hypocotyls of both species. Disparity between this current and previous studies might be related to our use of hypocotyls rather than leaves for analysis of light regulation. For most dicots, true leaves do not develop on etiolated seedlings (Wang *et al.*, 1992; Patel and Berry, 2008). However, the cotyledons from early seedlings have been shown to undergo light regulation for Rubisco and other photosynthetic genes (Berry *et al.*, 1985; 1990; Wang *et al.*, 1992; Patel and Berry 2008). In amaranth, these also show similar patterns of C_4_ development and cell specificity to leaves, but there are differences (Wang *et al.*, 1992). Leaves originate from vegetative meristems and undergo several stages of growth, development, and differentiation. Cotyledons present on early hypocotyls develop from cell divisions that occur during seed development, with no cell division and limited morphological development after germination. Thus, while hypocotyls provide an excellent system for studying light regulation and early C_4_ development, levels of Rubisco mRNA and protein production in these embryonically derived tissues may not necessarily correspond to those observed in mature C_3_ and C_4_ leaves.

### RLSB as a unique determinant of post-transcriptional *rbc*L expression and Rubisco accumulation

Plants that utilize C_4_ photosynthesis are critical for many agricultural and industrial applications, including food and biofuel production (Jones, 2011; Sage and Zhu, 2011; Von Caemmerer and Furbank, 2016; Sharwood *et al.*, 2016), as well as lesser known applications such as the bourbon distillation industry (Arnold and Simanek, 2016). Although characteristics of C_4_ plants have been known for decades, uncloaking the molecular basis of C_4_ photosynthesis remains an elusive goal (Hibberd and Covshoff, 2010; Berry et al., 2011, 2013, 2016; Langdale, 2011;Huang and Brutnell, 2016). Many studies have provided evidence that post-transcriptional regulation plays a role in photosynthetic gene expression (Berry *et al.*, 2011, 2013, 2016; Brown *et al.*, 2011; Garner *et al.*, 2016; Williams *et al.* 2016), and regulation of C_4_ genes at this level may be more significant than previously thought. In support of this hypothesis, a recent transcriptome study found post-transcriptional regulation of mRNA stability to be more prominent in C_4_ plants relative to C_3_ plants (Fankhauser and Aubry, 2017). The results presented here provide further evidence for RLSB as a unique mRNA-binding protein involved with *rbc*L gene expression in all plants, with stepwise modifications in its leaf localization correlating with the acquisition of BS cell-specific Rubisco production along the C_3_ to C_4_ species gradient. These modifications are superimposed on the basic shared characteristics of tissue-specific and light-mediated control, which were not altered during C_4_ evolution in this genus. As one of the few post-transcriptional regulatory components implicated in C_4_ expression, the ancient highly conserved RLSB protein may serve as a paradigm for the identification, functional characterization, and evolutionary analysis of such regulators as studies into the origins and processes of this essential photosynthetic pathway move forward.

## Supplementary data

Supplementary data are available at *JXB* online.

Fig. S1. Amino acid sequences of RLSB homologs in the genus *Flaveria*.

Fig. S2. Original unedited loading order of Fig. 6B.

Table S1. Transcriptome sequencing data of *Flaveria* in the NCBI database

Table S2. *Flaveria* transcriptome assembly.

Table S3. Three outgroup species from BLAST4OneK.

Table S4. List of primer sequences used for this study.

## Supplementary Material

supplementary_figures_S1_S2

supplementary_Table_S1

supplementary_Table_S2

supplementary_Table_S3

supplementary_Table_S4

## Abbreviations:

LSU: Rubisco large subunit protein
NAD-ME: NAD-dependent malic enzyme
PEPCase: phosphoenolpyruvate carboxylase
RLSB: *rbc*L RNA S1 binding domain protein
SSU: Rubisco small subunit protein;
UTR: untranslated region of mRNA.
