# Supplementary material for: An rbcL mRNA-binding protein is associated with C3 to C4 evolution and light-induced production of Rubisco in Flaveria
Source: J Exp Bot. 2017 Aug 8;68(16):4635–49. doi: 10.1093/jxb/erx264 (PMC5853808; doi:10.1093/jxb/erx264)

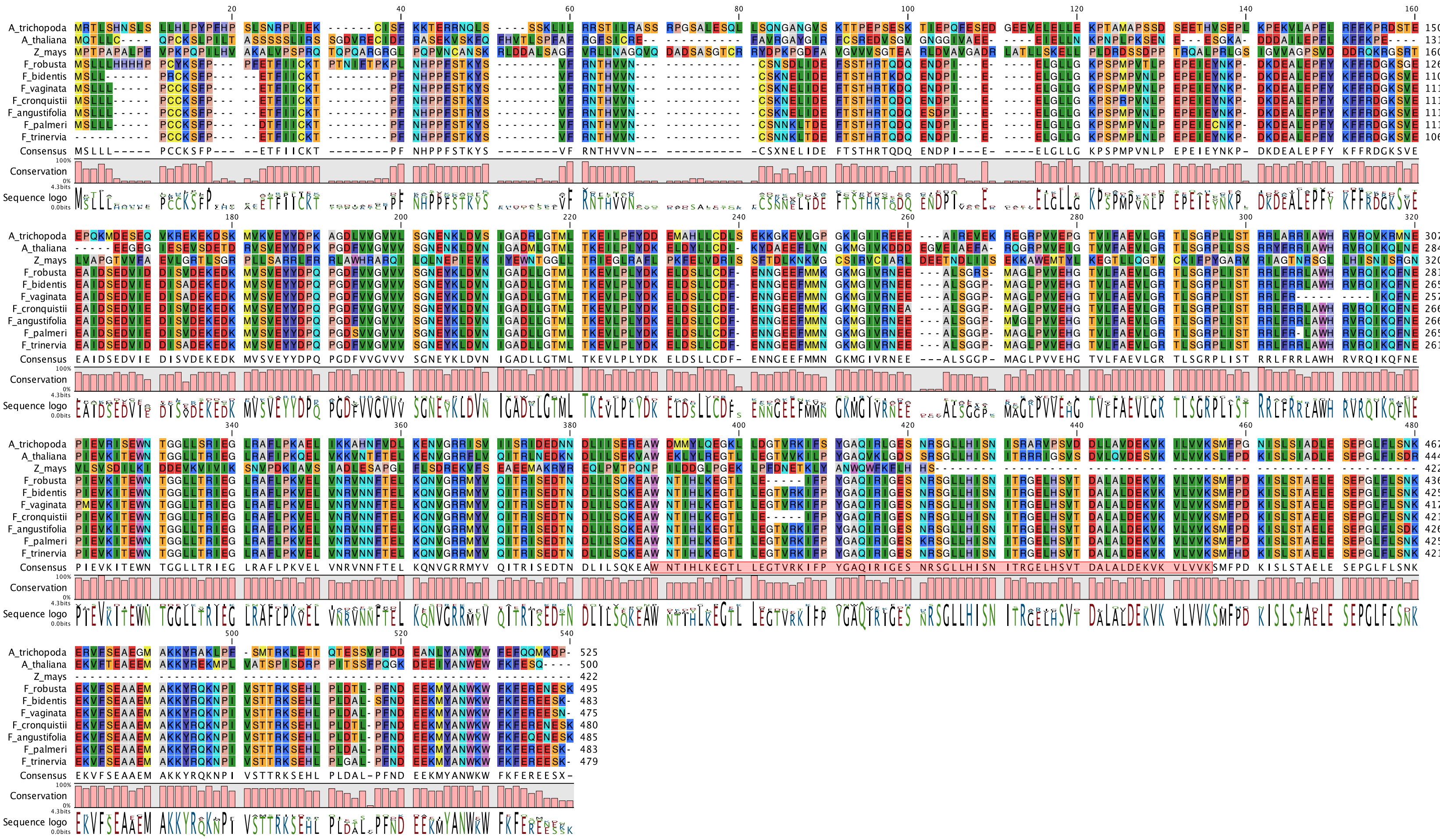

Fig. S1: Representative RLSB orthologs in the genus *Flaveria*. RLSB proteins are present and highly conserved within the genus *Flaveri*a. The translated amino acid sequences for RLSB orthologs in the seven *Flaveria* sequences were aligned using the MUSCLE multiple sequence algorithm (Edgar, 2004) implemented in the CLC Main Workbench 7.7.2. CLC Genomics Workbench 8.0.3 (https://www.qiagenbioinformatics.com/). Colors distinguish the different amino acid residues. The seven full length sequences used for this alignment were *F. robusta* (C_3) *F. bidentis* (C_4), *F. vaginata* (C_4-like), *F. cronquistii* (C_3), *F. angustifolia* (C_3-C_4/C_2), *F. palmerii* (C_4-like), and *F. trinerva* (C_4). The alignment shown here used the previously characterized RLSB from the C_3 dicot *Arabidopsis thaliana* (translated mRNA to protein) (Bowman et al., 2013) as a reference for comparison of the C_3, C_3-C_4, and C_4 dicot *Flaveria* sequences. Also included for comparison are sequences from *Zea mays*, a C_4 monocot, and *Amborella trichopda,* a basal angiosperm. The *Flaveria* sequences were used with permission from Dr. Julian Hibberd, Department of Plant Sciences, Cambridge University, Cambridge UK. Note that the conserved S1 binding domain occurs from position 390 to position 454, as indicated by a red highlight.

Fig. S2: Original unedited loading of Figure 6B. Note that the lanes in this figure are presented in their original loading order of Dark, Light, and Greening, instead of the rearranged order of Light, Dark, and Greening shown in Figure 6B. Other than the re-ordering described in the Fig. 6B legend, there were no changes made to the gel images shown.

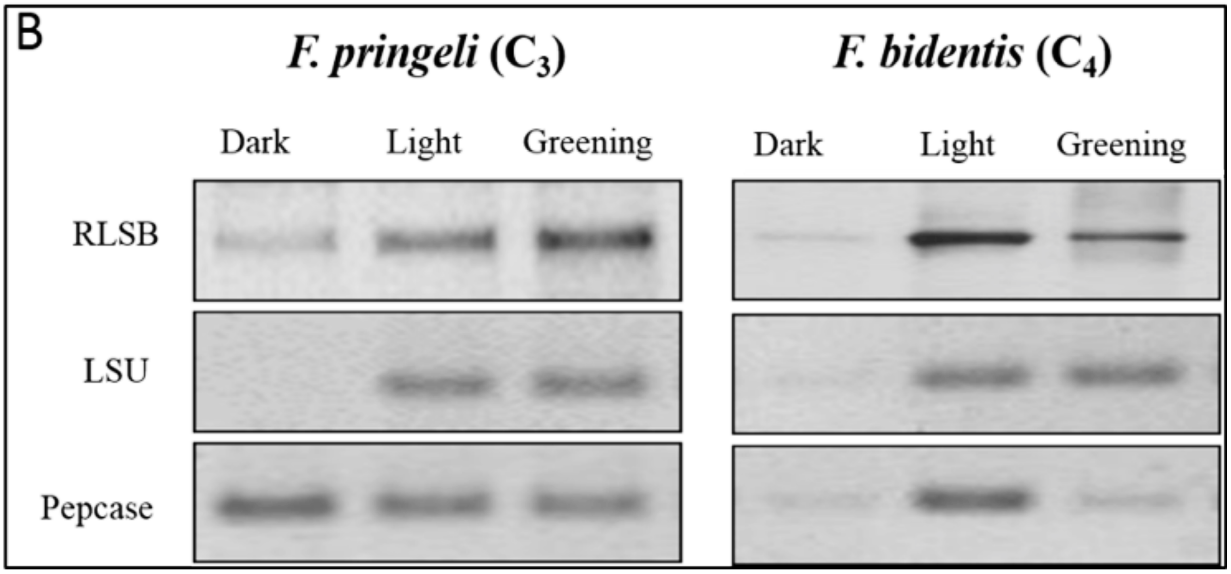

Supplement: supplementary_figures_S1_S2 [file erx264_suppl_supplementary_figures_s1_s2.pdf]
