# Supplementary material for: An rbcL mRNA-binding protein is associated with C3 to C4 evolution and light-induced production of Rubisco in Flaveria
Source: J Exp Bot. 2017 Aug 8;68(16):4635–49. doi: 10.1093/jxb/erx264 (PMC5853808; doi:10.1093/jxb/erx264)
Supplement: supplementary_Table_S4 [file erx264_suppl_supplementary_table_s4.pdf]

Table S4: List of primer sequences used for this study.

*F.bidentis rbc*L forward: AGTGTCTACGCGGTGGACTTGATT

*F.bidentis rbc*L reverse: TGCAGTCGCATTCAAGTAATGCCC

F.bidentis RLSB Fwd: TATTGGAGCTGATCTTCTTGG

F.bidentis RLSB Rev: AATAGTATTCCAAGCTTCCTTTTGAG

F.bidentis Actin Fwd: ACACTGTGCCAATCTACGAGGGTT

F.bidentis Actin Rev: ATTTCACGCTCTGCTGTTGTGGTG

F.pringlei RLSB Fwd: ATATTGGAGCTGATCTTCTTGGAAC

F.pringlei RLSB Rev: CCAATTAGCATACATCTTTTCTTCAT

*F.pringlei rbc*L forward: AGTGTCTACGCGGTGGACTTGATT

*F.pringlei rbc*L reverse: TGCAGTCGCATTCAAGTAATGCCC

F.pringlei Actin Fwd: ACACTGTGCCAATCTACGAGGGTT

F.pringlei Actin Rev: ATTTCACGCTCTGCTGTTGTGGTG
